# Supplementary figures and images for: Bimodal regulation of axonal transport by the GDNF-RET signalling axis in healthy and diseased motor neurons
Source: Cell Death Dis. 2022 Jul 7;13(7):584. doi: 10.1038/s41419-022-05031-0 (PMC9263112; doi:10.1038/s41419-022-05031-0)

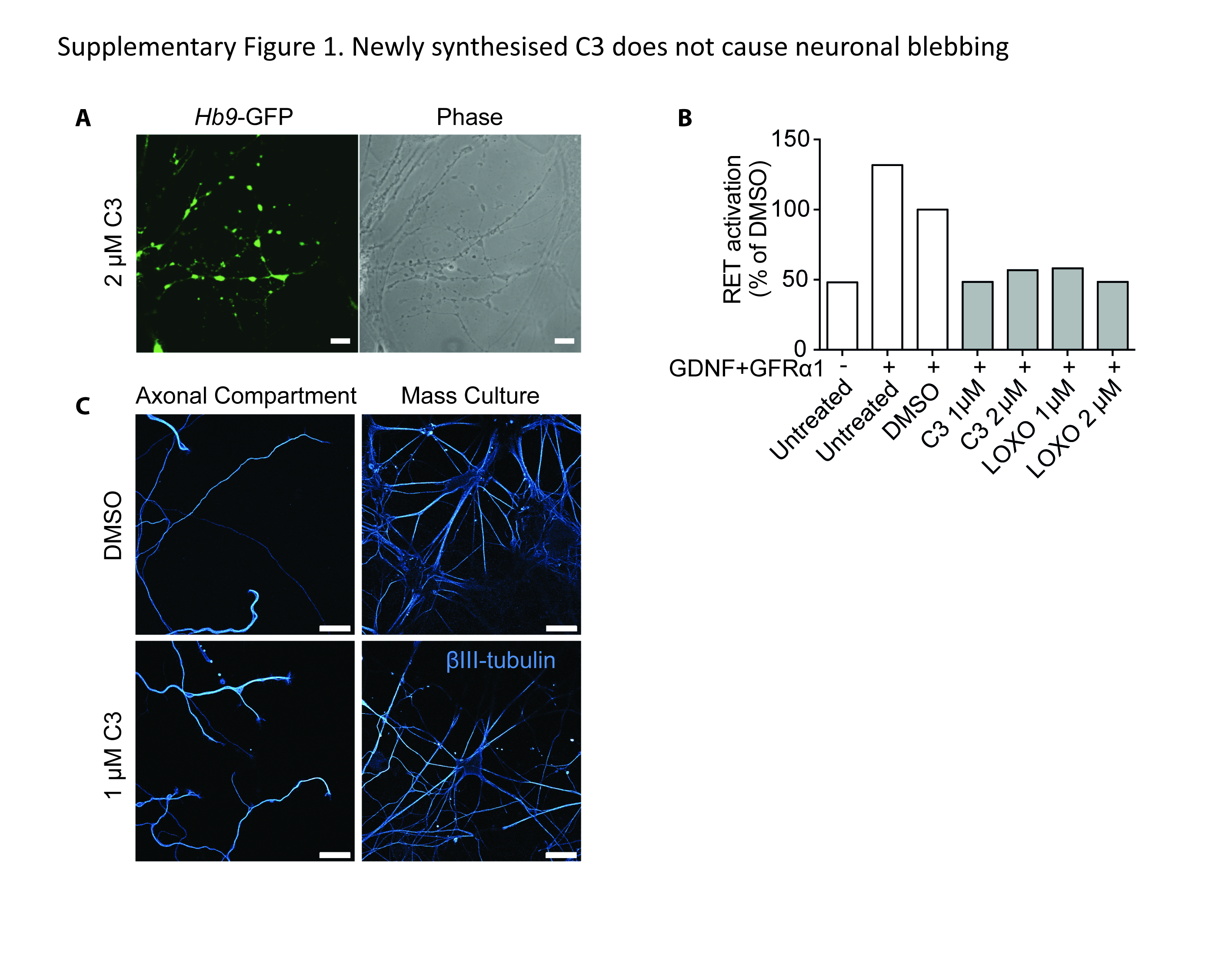

Supplement: Supplementary file 1 — Supplemental Figure 1 [file 41419_2022_5031_MOESM1_ESM.tif]

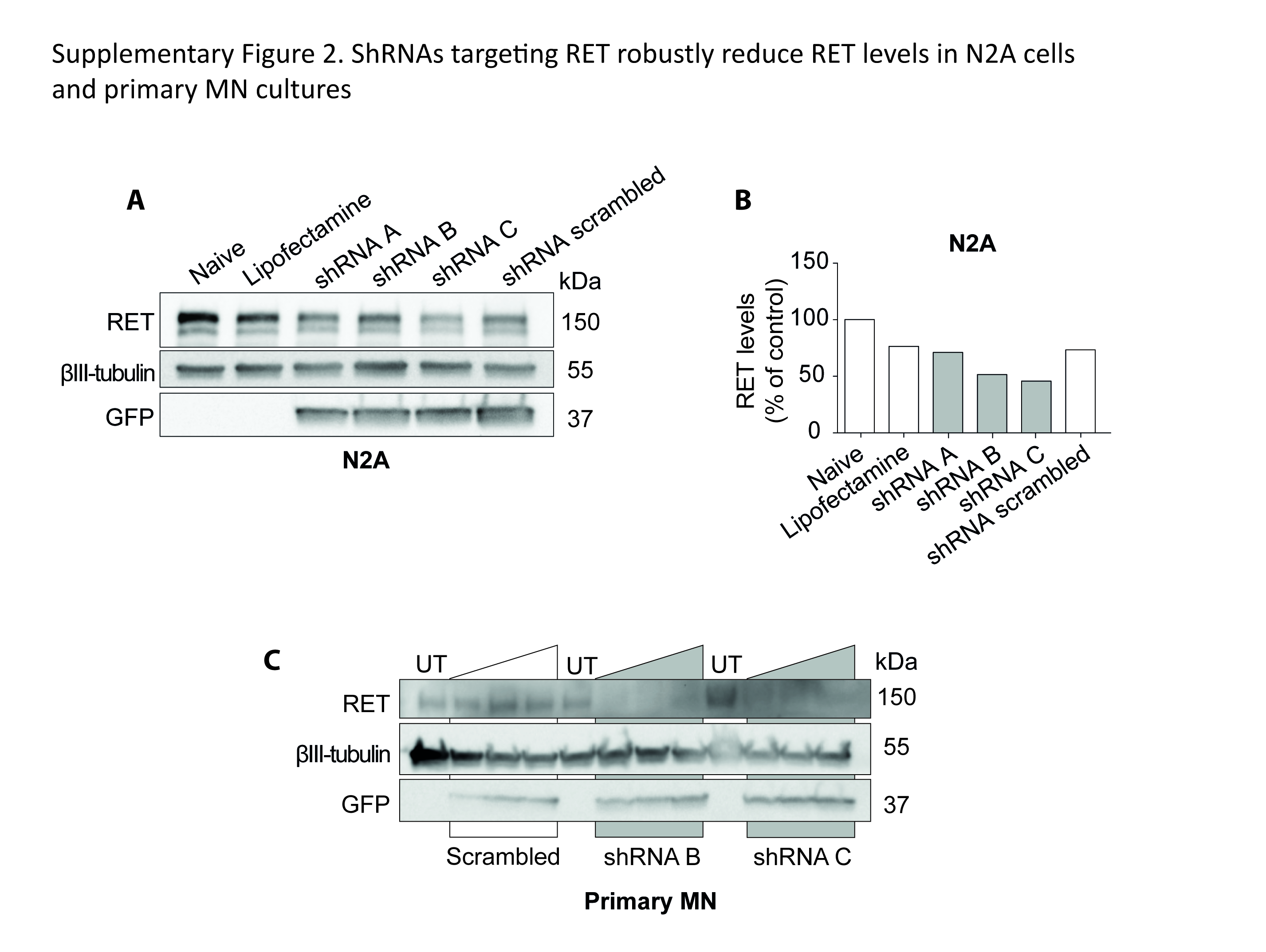

Supplement: Supplementary file 2 — Supplemental Figure 2 [file 41419_2022_5031_MOESM2_ESM.tif]

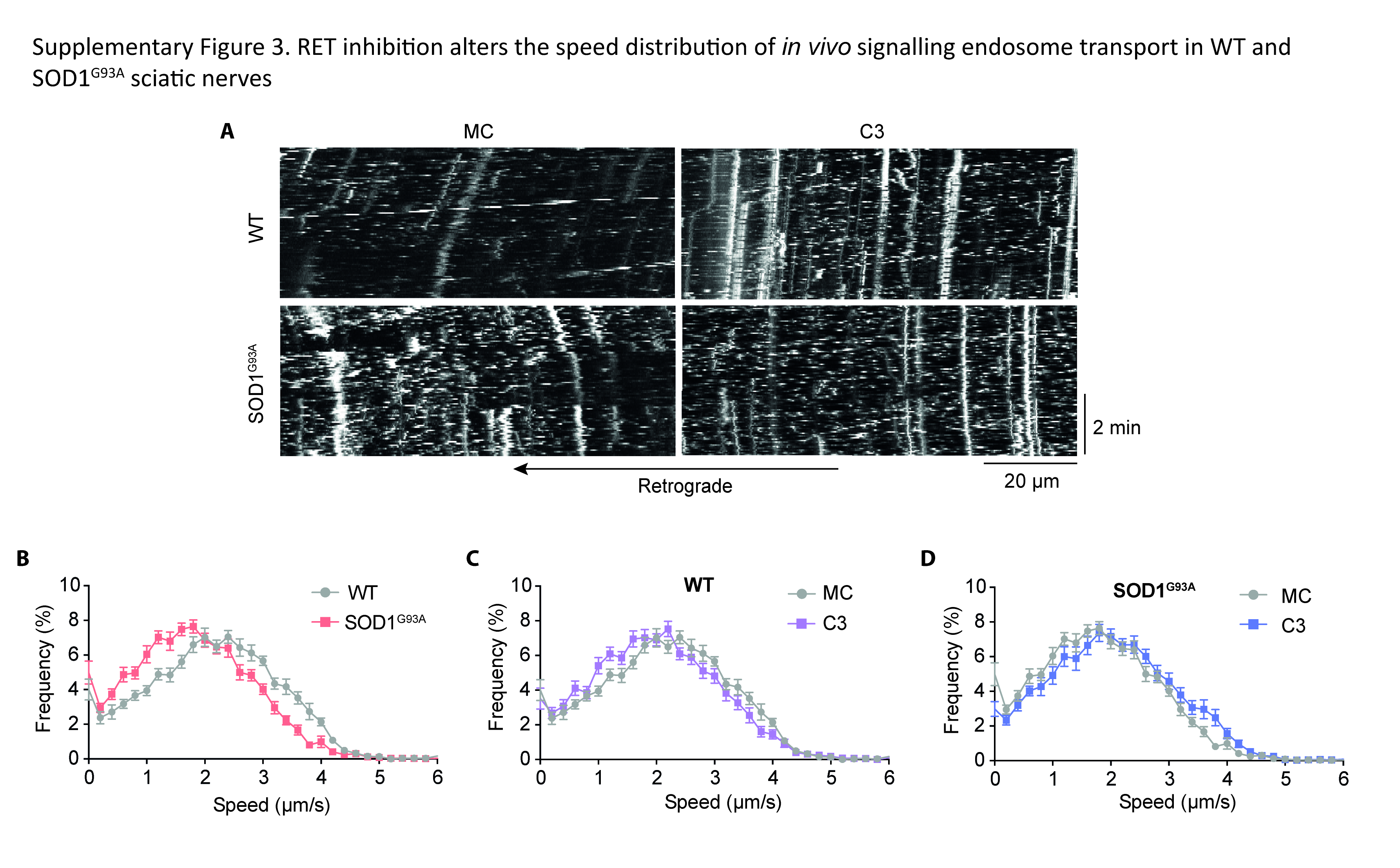

Supplement: Supplementary file 3 — Supplemental Figure 3 [file 41419_2022_5031_MOESM3_ESM.tif]

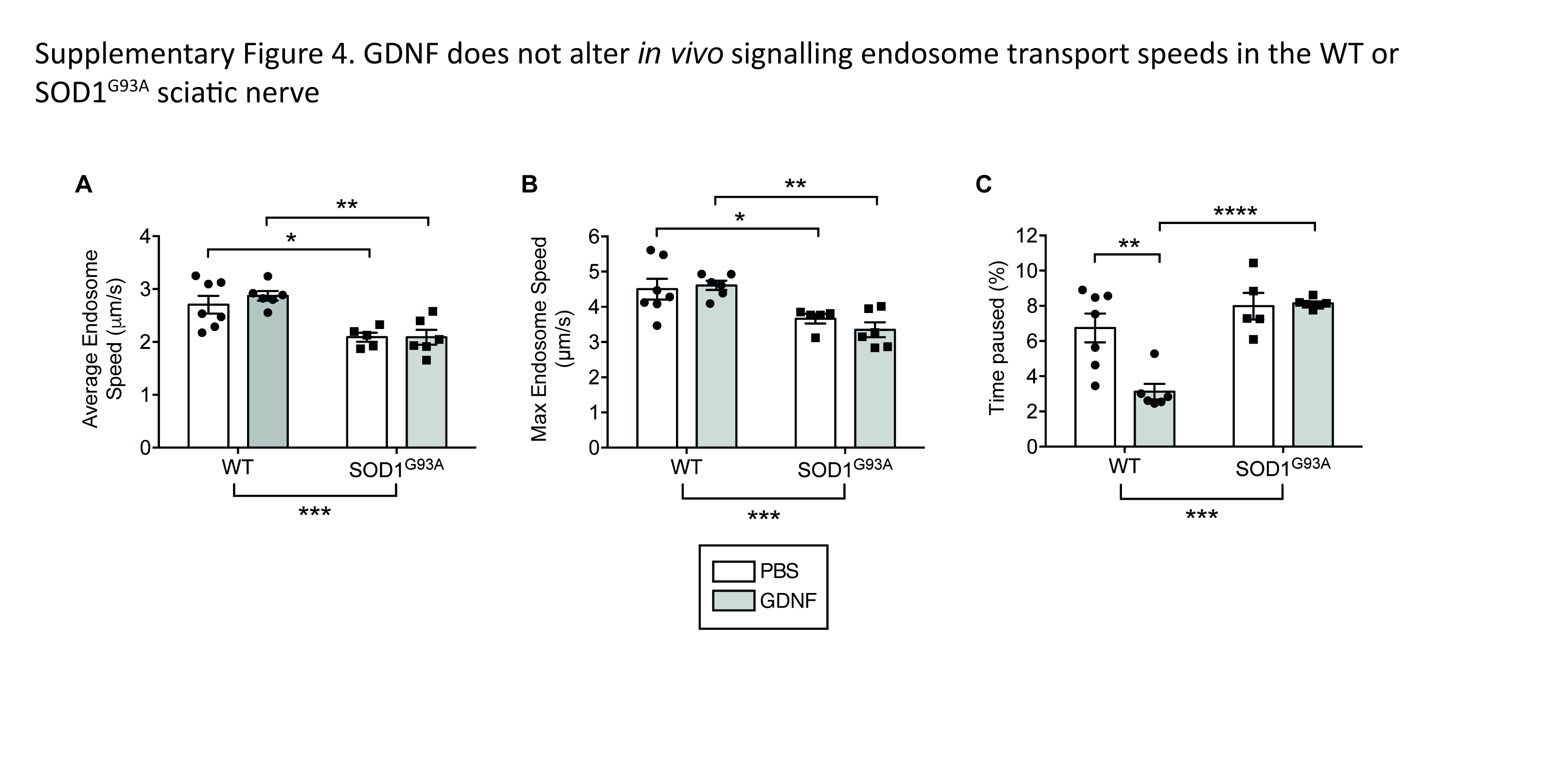

Supplement: Supplementary file 4 — Supplemental Figure 4 [file 41419_2022_5031_MOESM4_ESM.tif]

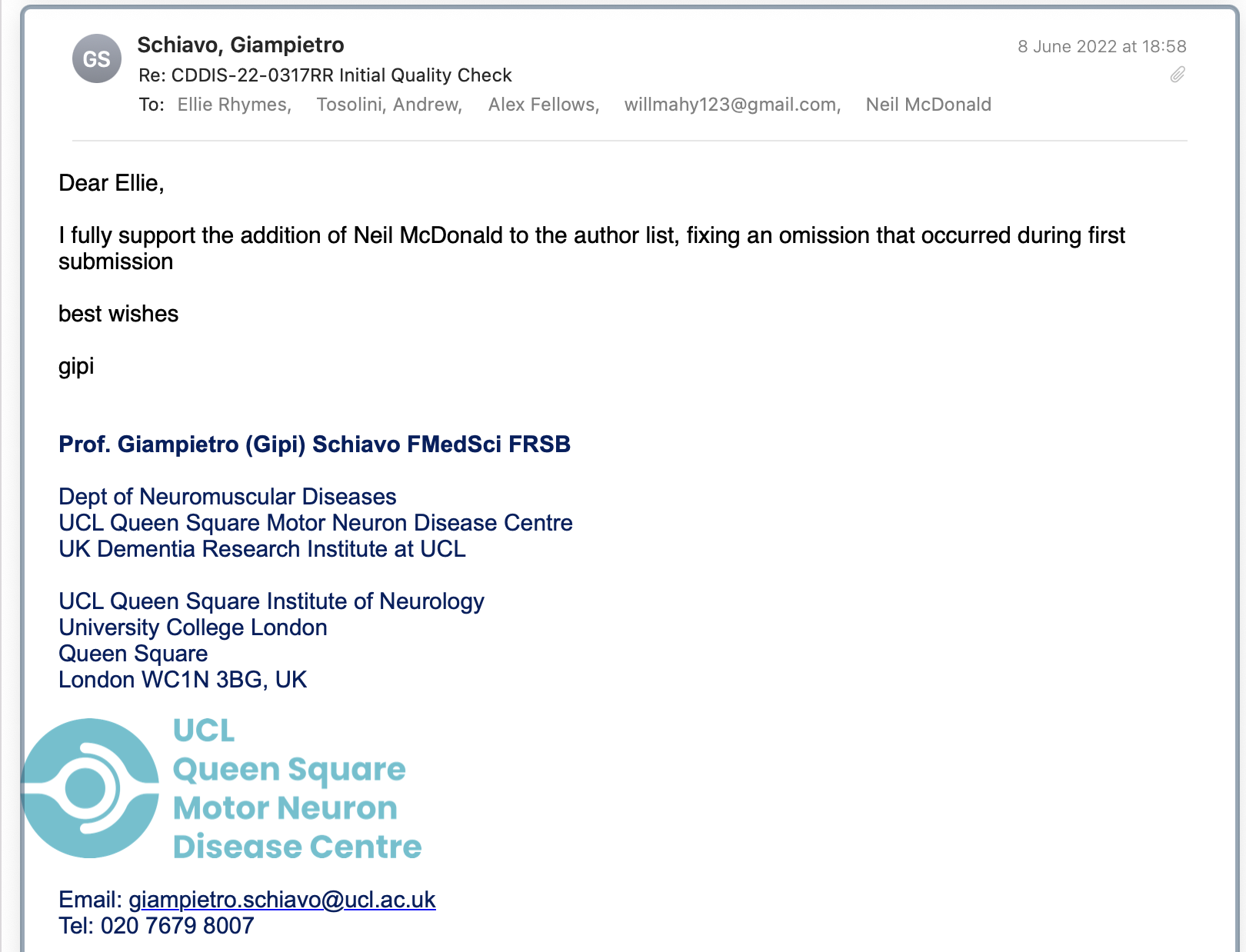

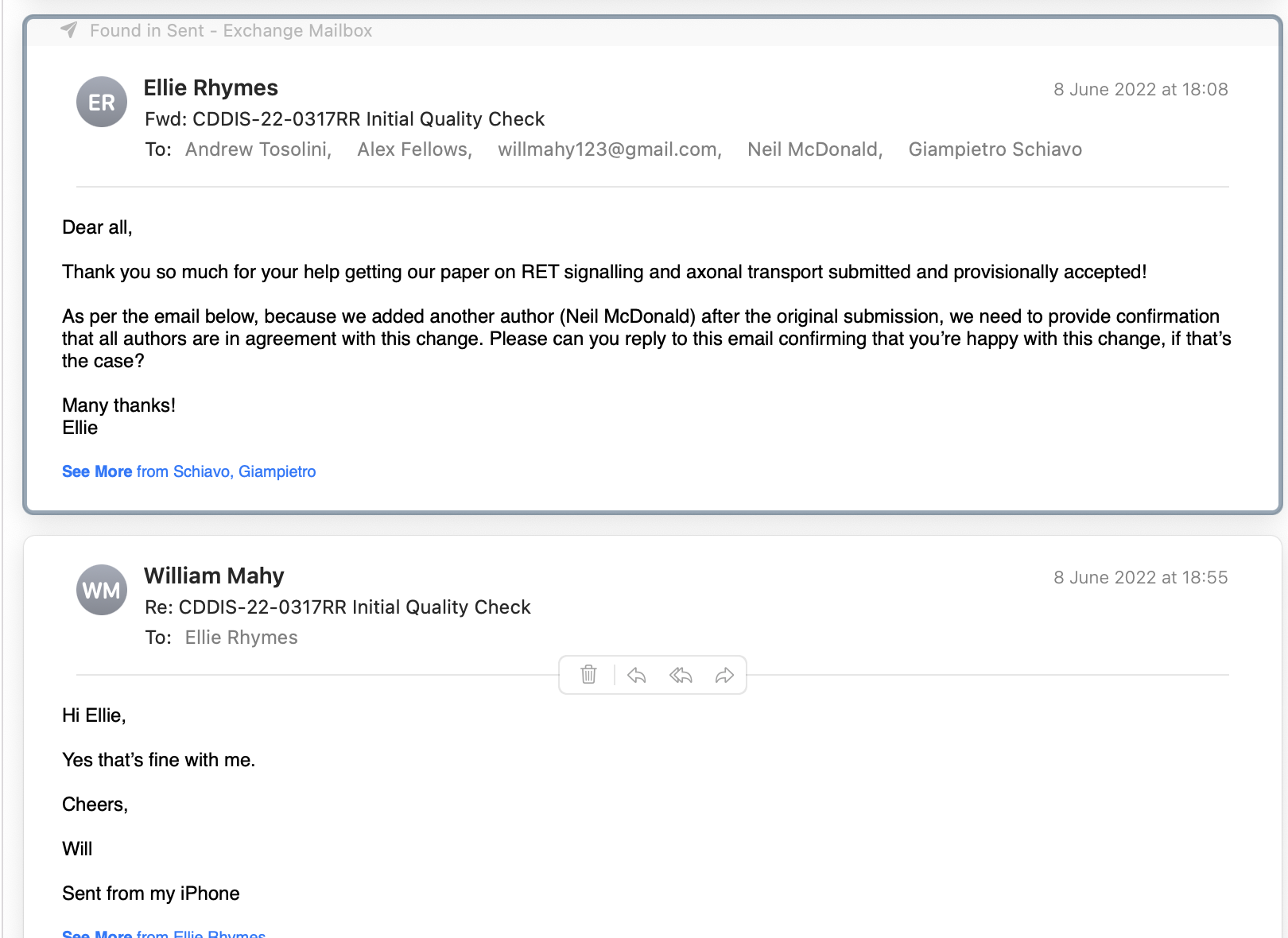


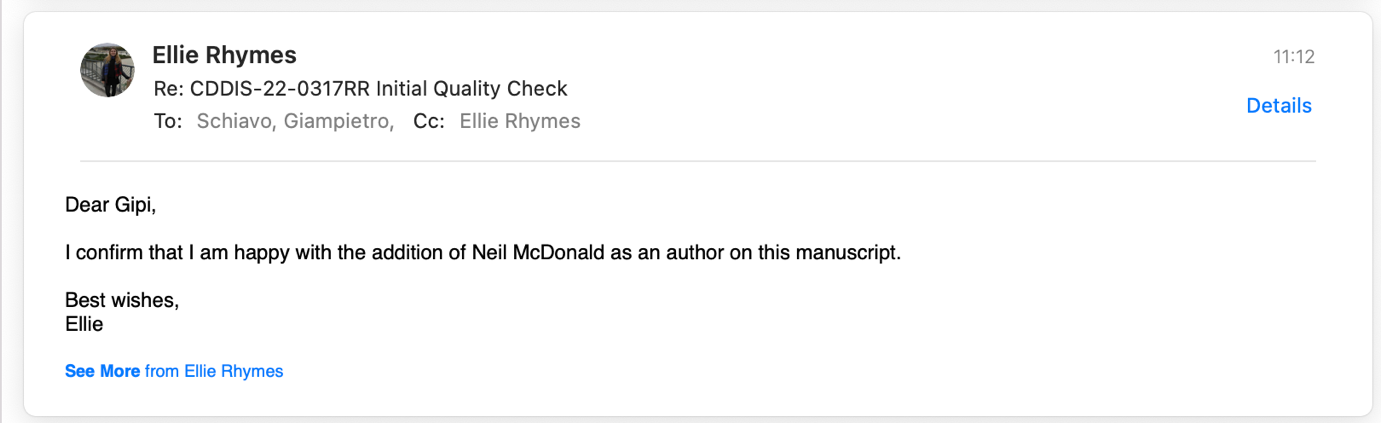


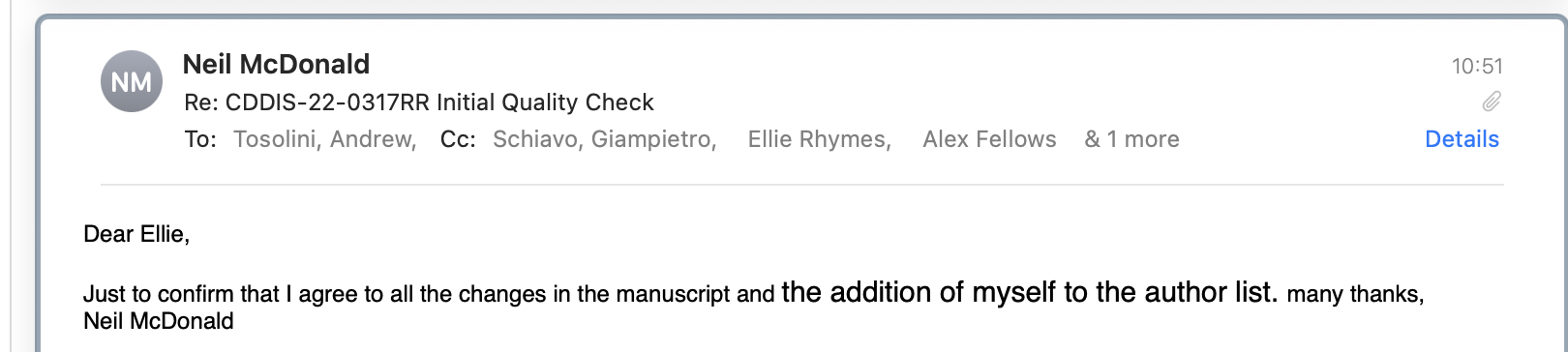


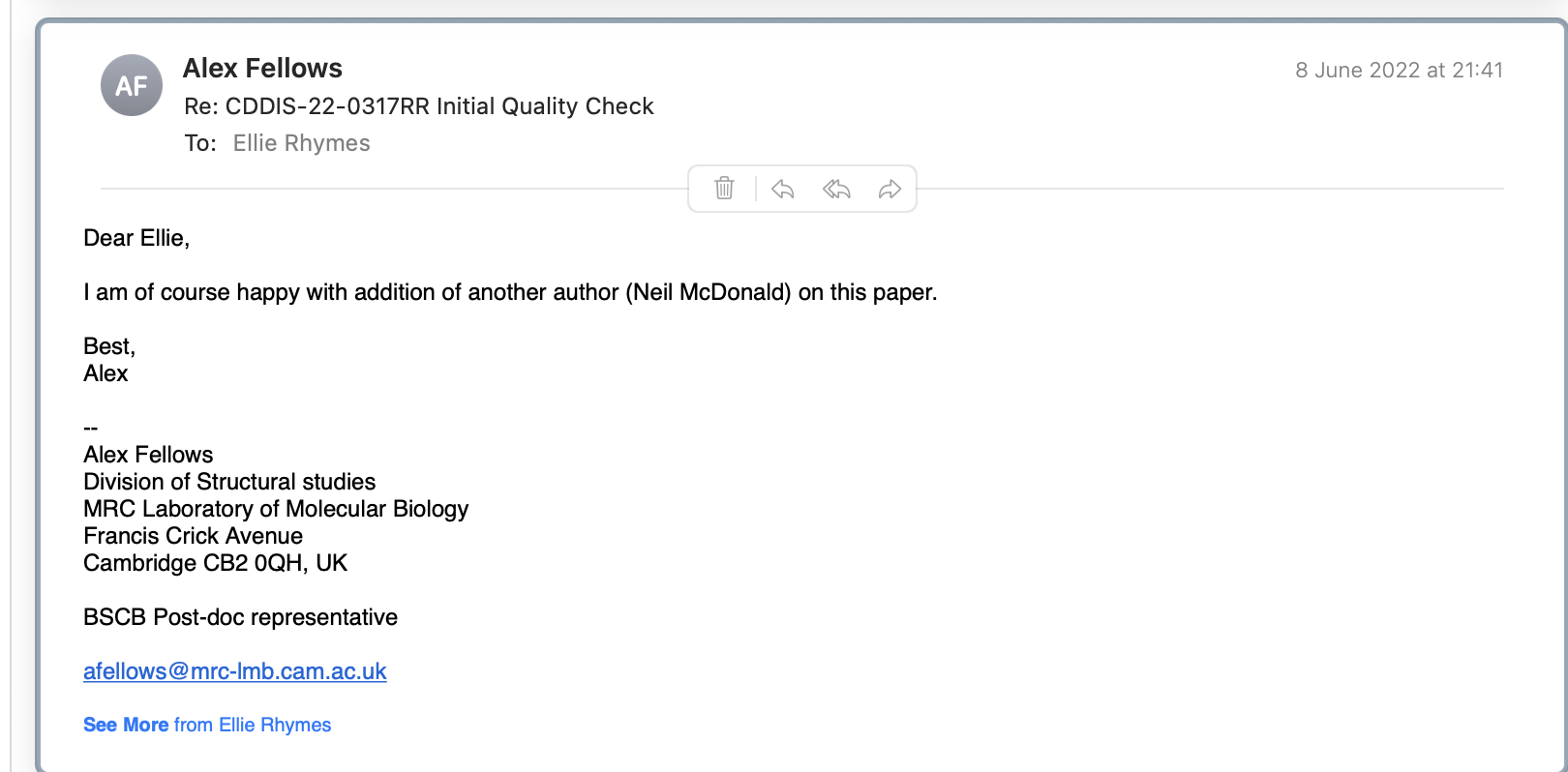


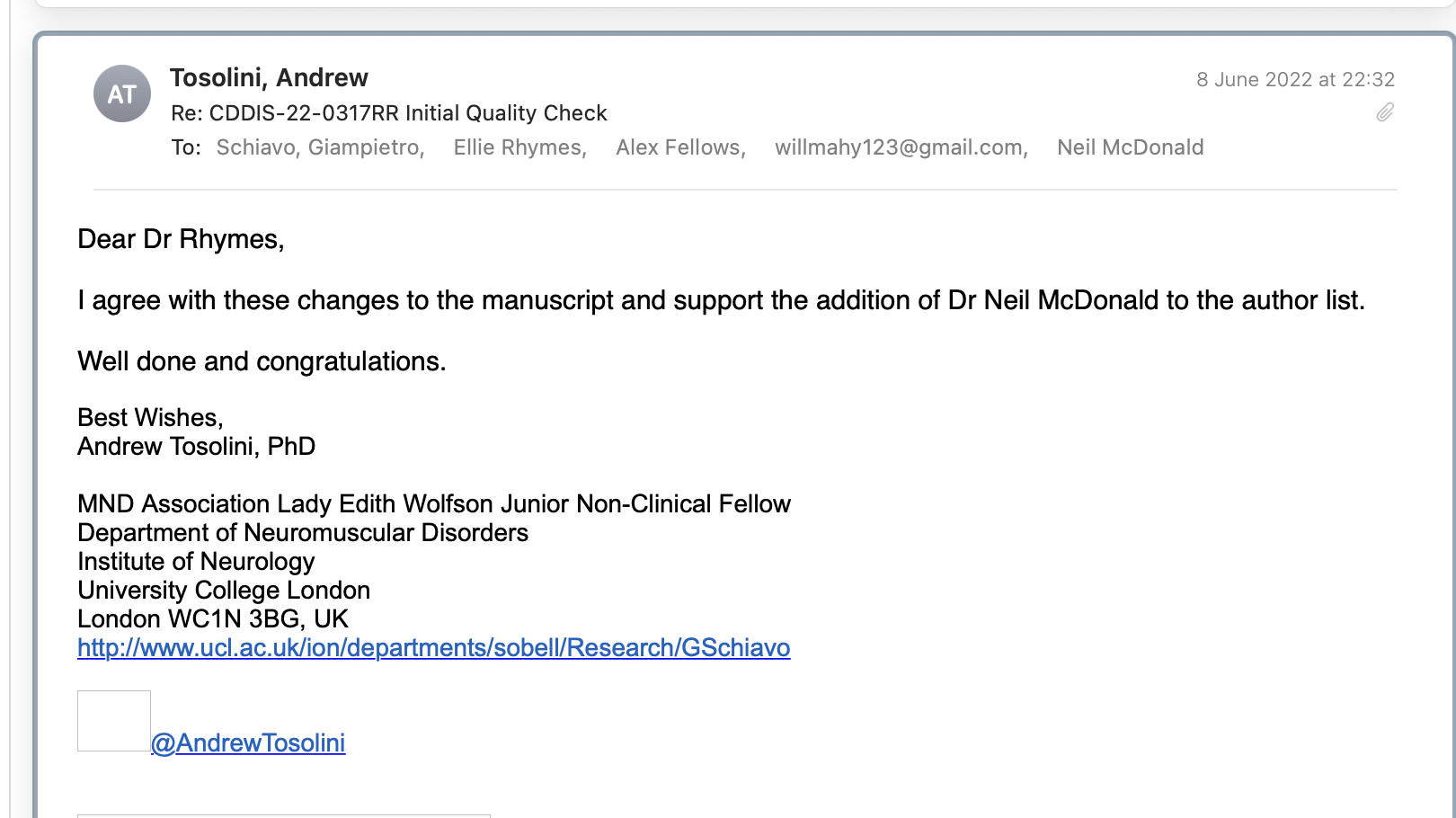

Supplement: Supplementary file 7 — Author list confirmation [file 41419_2022_5031_MOESM7_ESM.docx]
