# Supplementary material for: Bimodal regulation of axonal transport by the GDNF-RET signalling axis in healthy and diseased motor neurons
Source: Cell Death Dis. 2022 Jul 7;13(7):584. doi: 10.1038/s41419-022-05031-0 (PMC9263112; doi:10.1038/s41419-022-05031-0)
Supplement: Supplementary file 5 — Supplemental Figure Legends [file 41419_2022_5031_MOESM5_ESM.docx]

**Supplementary Figure legends**

**Supplementary Figure 1. Newly synthesised C3 inhibitor does not cause neuronal blebbing.**

1. *Hb9*-GFP and phase contrast images of ES-derived MNs after treatment with 2 µM of the original C3 compound used in the kinase inhibitor screen. Neurons were imaged 3-5 days after embryoid body dissociation. Scale bar represents 10 µm.
2. Quantification of RET activation after treatment with C3 and LOXO-292 observed in Fig.1B. RET activation was determined by normalising phospho RET band intensities to total RET, and is represented as a percentage of the average intensity of DMSO bands.
3. βIII-tubulin staining of mixed ventral horn cultures in MFCs or mass culture following treatment with 1 µM of the newly synthesised batch of C3 or DMSO for 1 h. Scale bars, 20 µm.

**Supplementary Figure 2. shRNAs targeting RET robustly reduce RET levels in N2A cells and primary MN cultures.**

1. RET, GFP and tubulin levels in N2A cell lysates after transfection with each RET-shRNA plasmid, and the scrambled control.
2. Quantification of RET knockdown in N2A cells, relative to naïve sample.
3. RET, GFP and tubulin levels in primary MN cultures after transduction with increasing volumes of lentiviral particles (2, 4 and 6 µl) expressing scrambled and RET-targeting shRNAs.

N = 1 biological replicate for preliminary optimization.

**Supplementary Figure 3. RET inhibition alter *in vivo* signalling endosome transport in the WT and SOD1^G93A^ sciatic nerves.**

1. Representative kymographs from axonal transport videos in WT and SOD1^G93A^ sciatic nerves.
2. WT vs SOD1^G93A^ speed distribution curves after treatment with control vehicle (1% MC) vs C3 inhibitor.
3. Speed distribution of signalling endosomes in WT sciatic nerve, comparing control vehicle vs C3 inhibitor.
4. Speed distribution of signalling endosomes in SOD1^G93A^ sciatic nerve, after treatment with control vehicle vs C3 inhibitor.

**Supplementary Figure 4. GDNF does not alter *in vivo* signalling endosome transport speeds in the WT or SOD1^G93A^ sciatic nerve.**

1. Average signalling endosome speed in the sciatic nerve of P73 WT and SOD1^G93A^ mice after intramuscular injection with 25 ng GDNF or vehicle control, PBS. GDNF had no significant effect on average endosome speeds (PBS:WT vs. GDNF:WT p = 0.61, PBS:SOD1^G93A^ vs. GDNF:SOD1^G93A^ p > 0.99). SOD1^G93A^ speeds were significantly slower than WT with both PBS and GDNF treatment (* p = 0.01 and * p = 0.003 respectively). Statistical tests: two-way ANOVA with Holm-Šídák’s multiple comparisons.
2. Maximum signalling endosome speed. GDNF had no significant effect on maximum endosome speed. Again, SOD1^G93A^ maximum speeds were significantly lower than WT with both PBS and GDNF treatment (* p = 0.0464 and * p = 0.00039 respectively, two-way ANOVA with Holm-Šídák’s multiple comparisons).
3. Signalling endosome pausing (%). GDNF significantly reduced signalling endosome pausing in the WT, but not SOD1^G93A^ sciatic nerve (** p = 0.0014). There was significantly more pausing in SOD1^G93A^ mice compared to WT with GDNF treatment (**** p < 0.0001), but no significant difference with PBS treatment (p = 0.3241). Statistical tests: two-way ANOVA with Holm-Šídák’s multiple comparisons.

In all analyses, there was a significant difference between genotypes (two-way ANOVA, *** p < 0.001). N = 6-7 animals. Points represent data from each individual animal. Bars represent mean ± SEM.
