# Supplementary material for: Bimodal regulation of axonal transport by the GDNF-RET signalling axis in healthy and diseased motor neurons
Source: Cell Death Dis. 2022 Jul 7;13(7):584. doi: 10.1038/s41419-022-05031-0 (PMC9263112; doi:10.1038/s41419-022-05031-0)
Supplement: Supplementary file 6 — Western blot originals [file 41419_2022_5031_MOESM6_ESM.pdf]

Figure 1B.

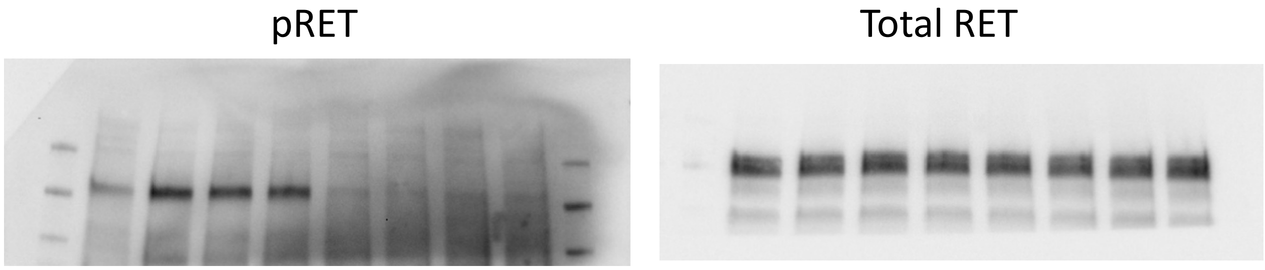

Figure 2G and 5A.

Phospho-blot

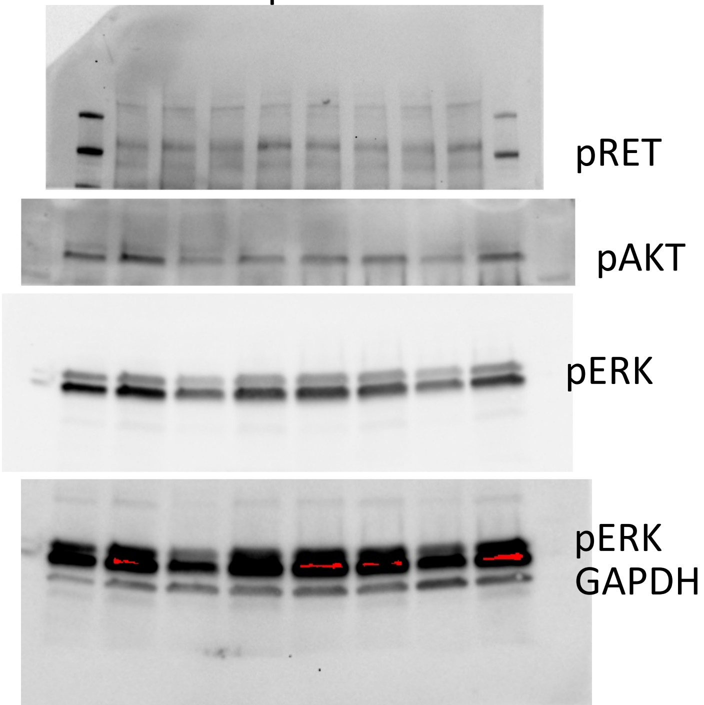

Blots were cut before primary antibody incubation to allow for staining of multiple proteins at once.

Figure 2G and 5A.

Total-blot

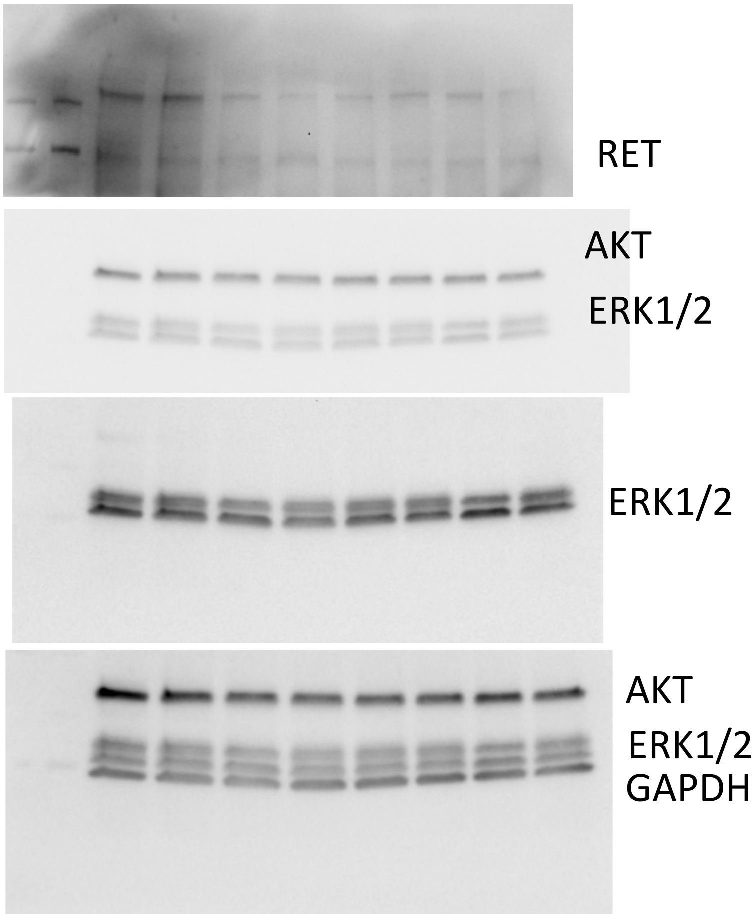

Figure 3F.

Phospho blot

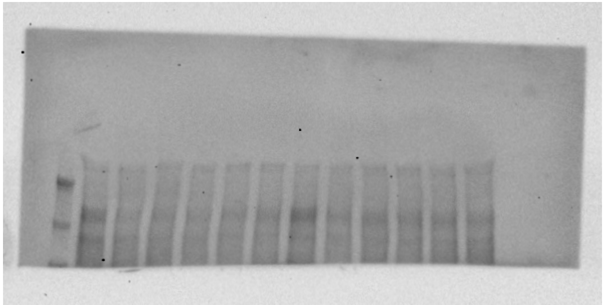

pRET

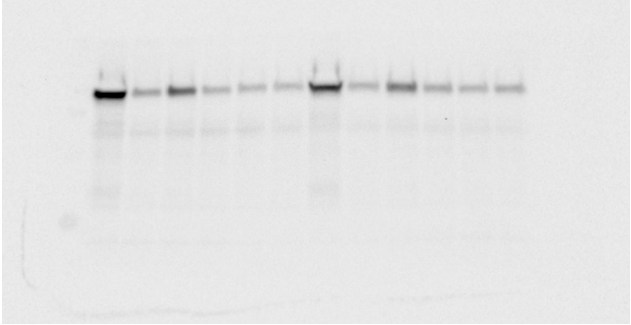

pAKT

pERK1/2

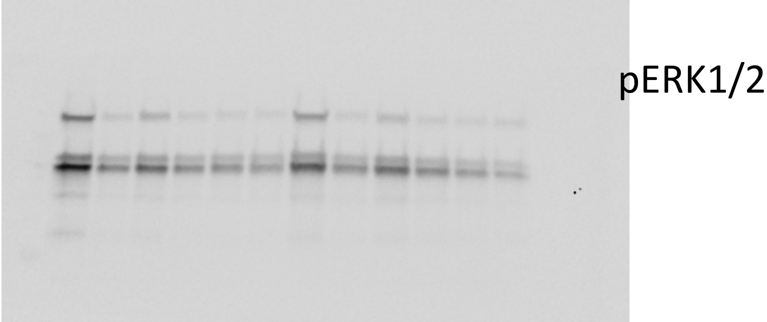

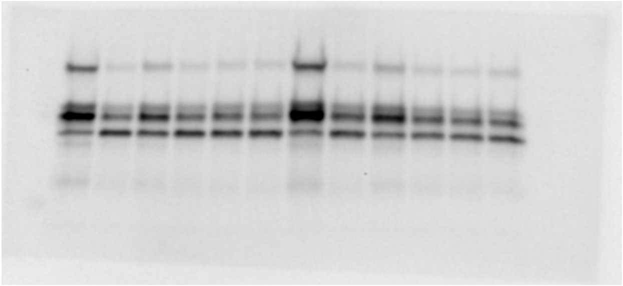

GAPDH

LHS is WT, shown in Figure 3F. RHS is the same experiment carried out in SOD1^{G93A} neurons.

Blots were cut before primary antibody incubation to allow for staining of multiple proteins at once.

Figure 3F.

Total blot

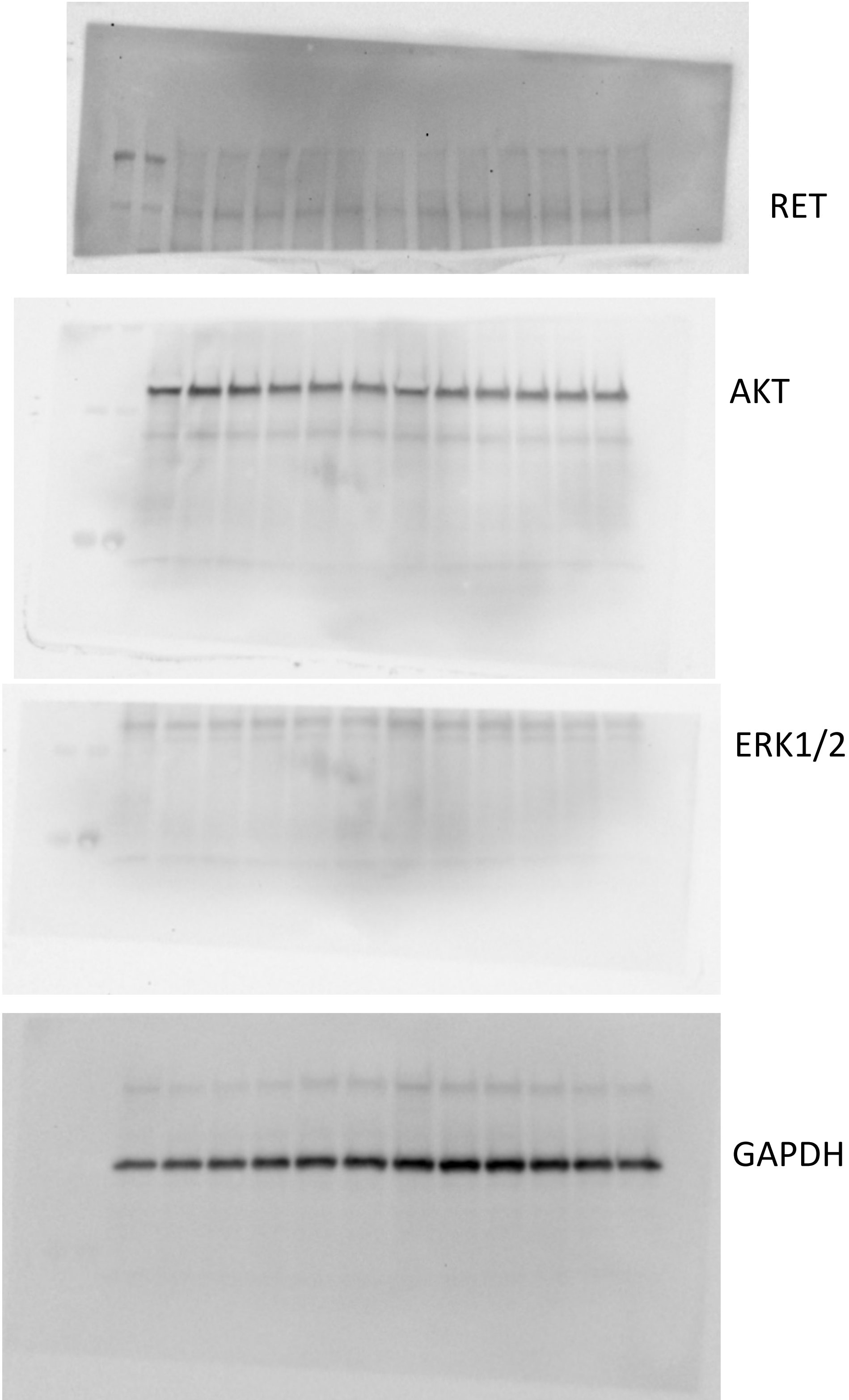

LHS is WT, shown in Figure 3F. RHS is the same experiment carried out in $SOD1^{G93A}$ neurons.

Blots were cut before primary antibody incubation to allow for staining of multiple proteins at once.

Figure 4C.

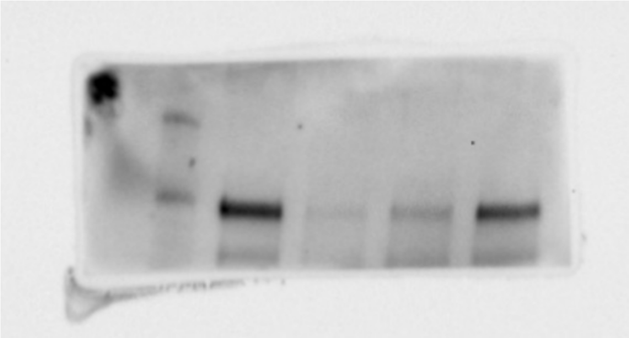

RET

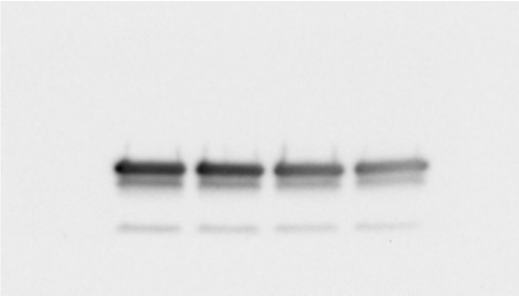

βIII-tubulin

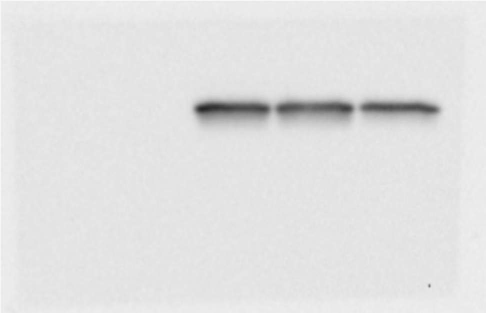

GFP

Blots were cut before primary antibody incubation to allow for staining of multiple proteins at once.

Supplementary Figure 2A.

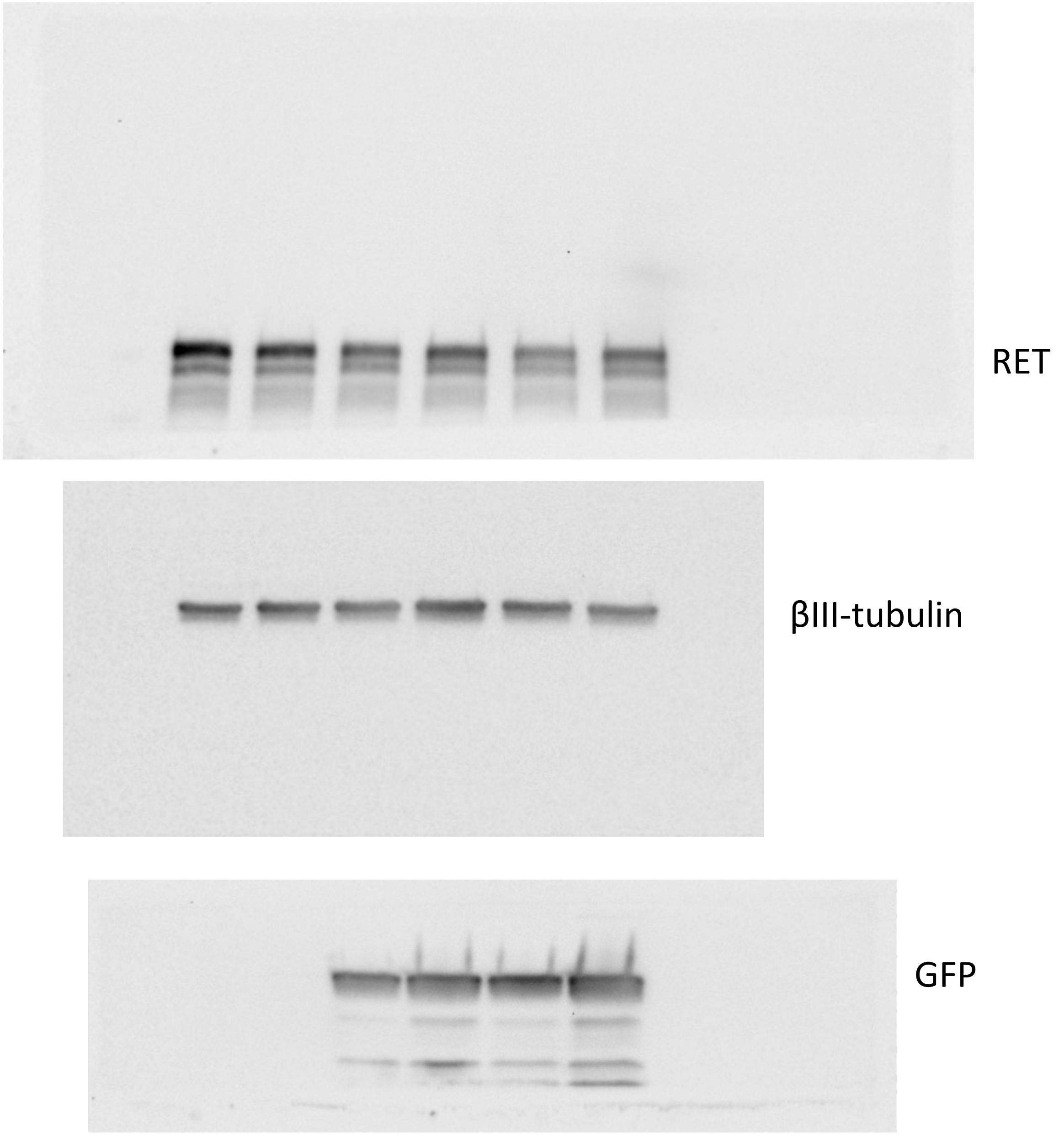

Blots were cut before primary antibody incubation to allow for staining of multiple proteins at once.

Supplementary Figure 2C.

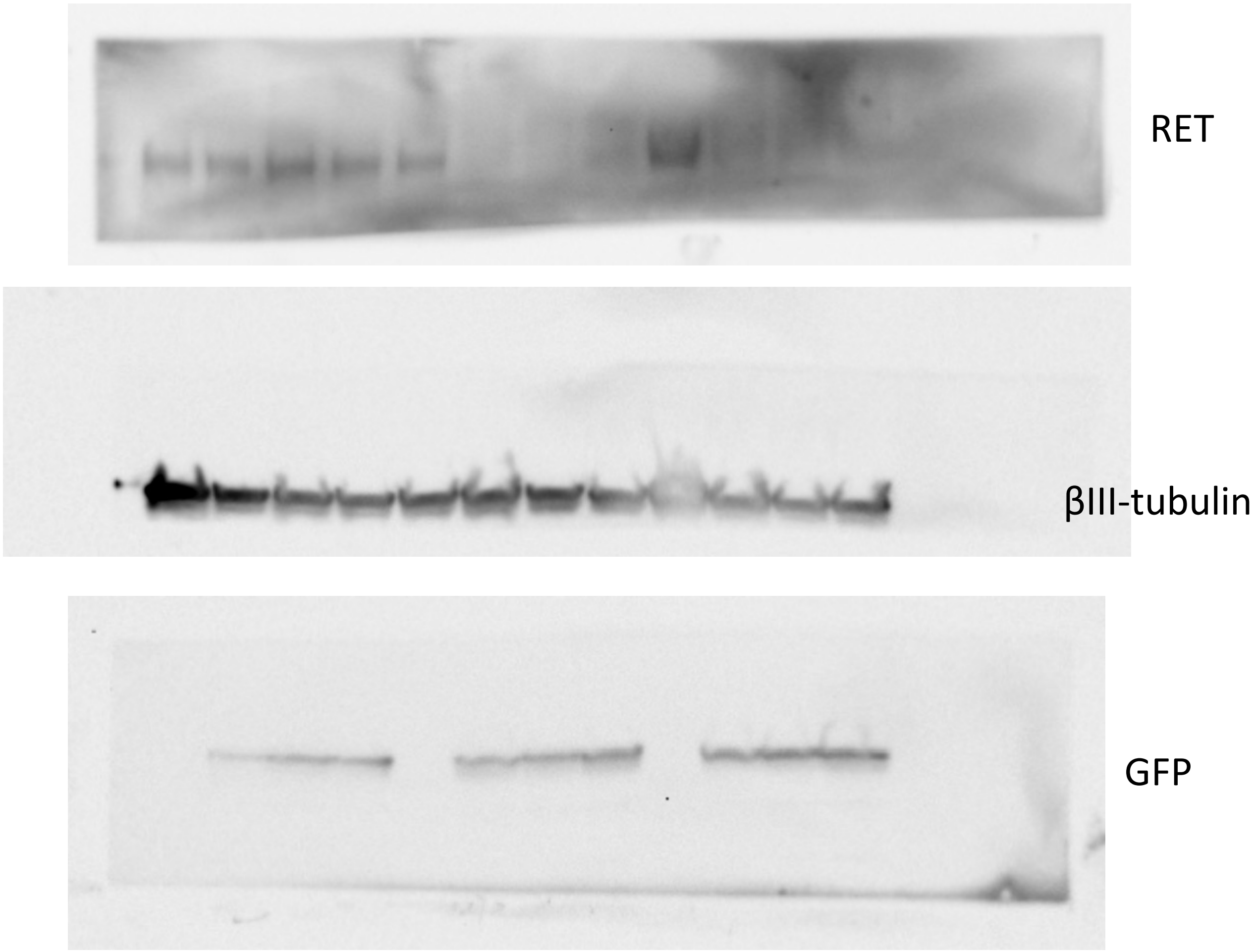

Blots were cut before primary antibody incubation to allow for staining of multiple proteins at once.
